# Microwave Power Absorption in Low-Reflectance, Complex, Lossy Transmission Lines

**DOI:** 10.6028/jres.112.015

**Published:** 2007-08-01

**Authors:** Jon Geist, Jayna J. Shah, Mulpuri V. Rao, Michael Gaitan

**Affiliations:** National Institute of Standards and Technology, Gaithersburg, MD 20899; George Mason University (GMU), Fairfax, VA 22030; National Institute of Standards and Technology, Gaithersburg, MD 20899

**Keywords:** absorption, microchannel, microfluidic device, microwave, transmission line

## Abstract

Simple sets of equations have been derived to describe the absorption of microwave power in three-region, lossy transmission lines in terms of S-parameter reflection and transmission amplitudes. Each region was assumed to be homogeneous with discontinuities at the region boundaries. Different sets of equations were derived to describe different assumptions about the amplitudes of the reflection coefficients at the different boundaries. These equations, which are useful when interference effects due to multiple reflections are small, were used to analyze S-parameter measurements on a transmission line that had a microfluidic channel in its middle region. The channel was empty for one set of measurements and filled with water for a second set of measurements. Most of the reflection assumptions considered here produced similar results for the fraction of the applied microwave power that was absorbed by a water-filled microchannel. This shows that the absorbed power is relatively insensitive to the reflection details as long as energy is conserved in the analysis. Another important result of this work is that the difference between the power absorbed in a water-filled channel and the power absorbed in the same empty channel can be a poor predictor of the power absorbed in the water in the presence of competing absorption processes such as absorption by the transmission-line metal.

## 1. Introduction

The possibility of microwave heating of lossy fluids in microfluidic devices with integrated transmission lines has been demonstrated recently [1, 2, 3, 4]. To optimize the performance of microwave heating in these types of devices, it will be necessary to distinguish between the absorption of microwave power in fluids in the microchannel and the absorption of microwave power in the metal components of the transmission line.

The standard way to calculate the power absorbed in a transmission line [5] requires knowledge of the real and imaginary components of the propagation con stants of the transmission lines connected to the transmission line being modeled. But it is possible that this type of information will not be available for transmission lines that are integrated with microfluidic devices. Therefore, it may be desirable to be able to analyze S-parameter measurements of this type of device without access to the type of data needed for the model described in [5].

In general, the reflection and transmittance spectra of a complex transmission line will have interference fringes due to inter-reflections between discontinuities in the transmission line. However, the fringes will be small perturbations on the spectra when all of the reflection coefficients are small, which is usually a design goal. In this case, it should be possible to ignore the phase information necessary to describe the amplitude and spacing of the interference fringes. The purpose of this paper is to develop approximations for the microwave power absorbed in lossy components of a three-region transmission line from S-parameter reflectance and transmittance amplitude measurements. The results should prove useful when the amplitude of the fringes on the absorption spectra are small compared to the effects of interest, as illustrated by application to experimental data on a real device.

Section 2 of this paper derives equations for the fractions of power reflected from, transmitted through, and absorbed in each region of a transmission line made up of three different homogeneous regions with a single reflective boundary. In other words, the reflection coefficients at all but one of the region boundaries are assumed to be zero. Section 3 derives equations for the same quantities when symmetric pairs of the four boundaries are equally reflective. These equations express the S-parameter data in terms of attenuation coefficients within the different regions of the transmission line and the reflection coefficients at the boundaries of the transmission-line regions. Section 4 inverts these equations to express the reflection and attenuation coefficients in terms of the S-parameter data. The resulting equations are then used to analyze experimental data from a three-region transmission line that contains a microfluidic channel in its middle region. Section 5 discusses the results and draws some conclusions.

## 2. Absorption in Transmission Lines With a Single Reflective Boundary

**Fig. 1:**
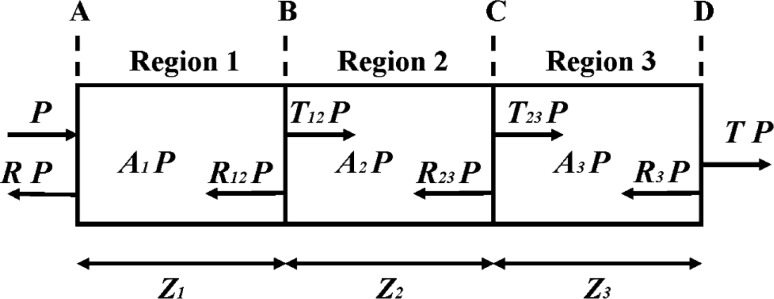
A complex generic transmission line consisting of three regions showing the power propagating in each direction at certain locations in the transmission line.

Figure 1 illustrates a complex generic transmission line that consists of three regions of length *z*_1_, *z*_2_, and *z*_3_, respectively, separated by boundaries labeled A, B, C, and D. The geometry and dielectric constants of all of the structures that make up each of the regions are assumed to be uniform within each region. It is also assumed that microwave power propagates along the transmission line with no loss due to emission of radiation. Finally, it is assumed that Regions 1 and 3 have identical microwave properties and the two transmission lines (not shown) to which the microwave probes are connected have identical microwave properties.

Let *P* be the power incident on Boundary A from the left, and let *RP* be the power leaving Boundary A to the left. Similarly, let *TP* be the power transmitted to the right from Boundary D. Now assume that *R* and *T*, which will be called the reflection and transmission, respectively, are the only experimental data available for the device of Fig. 1. Further assume that the real and imaginary parts of the propagation constants of the transmission lines (not shown) connected to the device shown in Fig. 1, as well as those of the three regions in that device, are not known. In this case, there is not enough information available to invert Maxwell’s equations to determine the propagation and attenuation constant in the regions of the device of Fig. 1 and in the transmission lines to which it is attached. This remains true even if Regions 1 through 3 are assumed to have identical properties and the transmission lines attached to the device in Fig. 1 are assumed to be identical.

In this case it is convenient to treat the transmission line in Fig. 1 as a power-flow problem [6]. A fraction *RP* of the incident power is reflected at Boundary A, a fraction *A*_1_*P* is absorbed in Region 1, a fraction *T*_12_*P* is transmitted across Boundary B, and a fraction *R*_12_*P* is also reflected at Boundary B. Similar descriptions apply to Regions 2 and 3, and, finally, a fraction *TP* is transmitted out the right side of the transmission line at Boundary D. By conservation of energy, *A* = *A*_1_ + *A*_2_ + *A*_3_ = 1 − *R* − *T* is the fraction of the power incident on the transmission line that is absorbed by the transmission line. *R* and *T* can be obtained directly from S-parameter measurements, and *A* can be calculated from *R* and *T*.

On the other hand, *A*_1_, *A*_2_, and *A*_3_ are complex functions of the microwave power-reflection coefficients and the phase differences between the microwave amplitudes reflected at and transmitted across the different boundaries. In general, these quantities are described by Maxwell’s equations. However, if interference between multiply reflected waves in the transmission line introduces only small effects (fringes on the absorption spectra), compared to the effects of interest, then simpler descriptions are possible. These are based on ray tracing, knowledge of the general nature of the solutions of Maxwell’s equations in homogeneous lossy regions, conservation of energy, and a number of independent reflection and transmission measurements equal to or greater than the number of unknown parameters. This idea is developed in the following two sections.

### 2.1 Reflection Only at Boundary A

First assume that the power-reflection coefficients at Boundaries B, C, and D are all zero. Then *R*_12_ = *R*_23_ = *R*_3_ = 0. In this case,

$$T=[1-\rho ]\tau_{1}\tau_{2}\tau_{3}=10^{\left(S_{21}/10\right)},$$ (1)

$$R=\rho =10^{\left(S_{11}/10\right)},$$ (2)

$$A_{1}=[1-\rho ]\left[1-\tau_{1}\right],$$ (3)

$$A_{2}=[1-\rho ]\tau_{1}\left[1-\tau_{2}\right],$$ (4)

$$A_{3}=[1-\rho ]\tau_{1}\tau_{2}\left[1-\tau_{3}\right],$$ (5)

$$\tau_{n}=\exp\left(-2\alpha_{n}z_{n}\right).$$ (6)

In these equations, *S*_11_ and *S*_21_ are the respective reflection and transmission coefficients in dB, *ρ* is the power-reflectance coefficient *S*_11_ at Boundary A, and *α_n_* for *n* = 1, …, 3 are the amplitude-attenuation coefficients for Regions 1–3, respectively. Even though the order of the factors in these equations is not important, it simplifies tracing the flow of power through the device.

The amplitude absorption coefficient of a material, which is often denoted by *α*, should not be confused with the amplitude attenuation coefficient *α_n_* of Region *n* of the transmission lines being considered here. Two times the amplitude absorption coefficient of a material, which can be calculated from the complex dielectric constant of that material, describes the power attenuation per unit distance of a collimated wave of electromagnetic radiation propagating through a homogeneous slab of that material. On the other hand, two times *α_n_* describes the power attenuation per unit distance of a guided wave of radiation propagating in Region *n* of a complex transmission line. In general *α_n_* will depend upon both the geometry and the frequency-dependent complex dielectric constants of all of the structures that make up Region *n* of the transmission line.

### 2.2 Reflection Only at Boundary B

Only when all but one of the reflection coefficients are zero are simple equations like Eqs. (1–5) rigorously true without regard to the phase relations of the microwave amplitudes. For instance, let *ρ* be the power reflection coefficient at Boundary B, and let all of the other reflection coefficients be zero. In this case, Eq. (2) and Eq. (3) must be replaced by

$$R=\tau_{1}\rho \tau_{1},$$ (7)

$$A_{1}=\left[1-\tau_{1}\right]+\tau_{1}\rho \left[1-\tau_{1}\right],$$ (8)

while the other equations remain valid.

### 2.3 Reflection Only at Boundary C

If *ρ* is the power reflection coefficient at Boundary C and all of the other reflection coefficients are zero, then Eqs. (2–4) must be replaced by

$$R=\tau_{1}\tau_{2}\rho \tau_{2}\tau_{1},$$ (9)

$$A_{1}=\left[1-\tau_{2}\right]+\tau_{1}\tau_{2}\rho \tau_{2}\left[1-\tau_{1}\right],$$ (10)

$$A_{2}=\tau_{1}\left[1-\tau_{2}\right]+\tau_{1}\tau_{2}\rho \left[1-\tau_{2}\right].$$ (11)

### 2.4 Reflection Only at Boundary D

Continuing in the same vein, if *ρ* is the power reflection coefficient at Boundary D, and all of the other reflection coefficients are zero, then Eq. (1) is still valid, but Eqs. (2–5) must be replaced by

$$R=\tau_{1}\tau_{2}\tau_{3}\rho \tau_{3}\tau_{2}\tau_{1},$$ (12)

$$A_{1}=\left[1-\tau_{1}\right]+\tau_{1}\tau_{2}\tau_{3}\rho \tau_{3}\tau_{2}\left[1-\tau_{1}\right],$$ (13)

$$A_{2}=\tau_{1}\left[1-\tau_{2}\right]+\tau_{1}\tau_{2}\tau_{3}\rho \tau_{3}\left[1-\tau_{2}\right],$$ (14)

$$A_{3}=\left[1-\tau_{1}\right]+\tau_{1}\tau_{2}\tau_{3}\rho \left[1-\tau_{3}\right].$$ (15)

## 3. Absorption in Transmission Lines With Two Reflective Boundaries

In the case where the amplitude of any two of the reflection coefficients is non-zero, it is still possible to derive good approximations to the reflectance and transmittance based on power flow, if the effects of interference are small compared to the effects of interest. Practically speaking, this means that the reflectance coefficients will be small compared to one, which is equivalent to requiring good impedance matching at all boundaries. When the impedance matching is poor, there will be large fringes on the *S*_11_ and *S*_21_ spectra due to interference between multiply reflected waves propagating on the transmission line. On the other hand, when the impedance matching is good, the fringes will be barely visible compared to the effects of interest in the spectra.

In the approach used here [6], the powers in multiply reflected waves are simply added, which washes out all interference effects and produces a result intermediate between the envelope of the fringe maxima (constructive interference) and the envelope of the fringe minima (destructive interference) in the S-parameter measurements. If the interference effects are large compared to the effects of interest, then the errors introduced by this approximation will be large. On the other hand, when the fringes are small compared to the effects of interest, then the derived equations will provide useful approximations.

### 3.1 Reflection Only at Boundaries A and D

Continue to assume that Regions 1 and 3 have identical properties and that *ρ* is the power reflection coefficient at each probe. To derive a general expression, it is necessary to have expressions for the reflectance of the transmission line at the left probe and trans mittance of the transmission line at the right probe. These can be derived [6] by expressing *A*_l_, *A*_2_, and *A*_3_ as well as *R* and *T* as an infinite series of terms; each term describing the power in a wave that has been multiply reflected a different number of times, and summing the series analytically, to produce closed-form expressions:

$$R=\frac{\rho \left[1+\tau^{2}(1-2\rho )\right]}{1-\rho^{2}\tau^{2}}\approx \rho \left[1+\tau^{2}\right],$$ (16)

$$T=\frac{{\left(1-\rho \right)}^{2}\tau }{1-\rho^{2}\tau^{2}}\approx [1-2\rho ]\tau ,$$ (17)

$$A_{1}=\frac{[1-\rho ]\left[1-\tau_{1}\right]\left[1+\tau_{1}\tau_{2}\tau_{3}\rho \tau_{3}\tau_{2}\right]}{1-\rho^{2}\tau^{2}}\approx \left[1-\tau_{1}\right]\left[1-\rho +\tau_{1}\tau_{2}\tau_{3}\rho \tau_{3}\tau_{2}\right],$$ (18)

$$A_{2}=\frac{[1-\rho ]\tau_{1}\left[1-\tau_{2}\right]\left[1+\tau_{2}\tau_{3}\rho \tau_{3}\right]}{1-\rho^{2}\tau^{2}}\approx \left[1-\tau_{2}\right]\tau_{1}\left[1-\rho +\tau_{2}\tau_{3}\rho \tau_{3}\right],$$ (19)

$$A_{3}=\frac{[1-\rho ]\tau_{1}\tau_{2}\left[1-\tau_{3}\right]\left[1+\tau_{3}\rho \right]}{1-\rho^{2}\tau^{2}}\approx \left[1-\tau_{3}\right]\tau_{1}\tau_{2}\left[1-\rho +\tau_{3}\rho \right],$$ (20)

$$\tau =\tau_{1}\tau_{2}\tau_{3}.$$ (21)

The approximations on the right hand side of Eq. (16) and Eq. (20) were chosen to conserve energy as well as be accurate to order *ρ* ^2^. They will he useful when *ρ* ^2^ << 1. They could also have been derived by ray tracing [6] to generate all terms linear in *ρ* without recourse to the sum of the series of all ray-tracing terms.

### 3.2 Reflection Only at Boundaries B and C

As another alternative reflection model, assume again that Regions 1 and 3 have identical properties, but *ρ* is the power reflection coefficient at Boundaries B and C of Region 2. In this case, the only expressions that need to be derived by the procedure described above are those for *R*_12_ and *T*_23_, because *A*_l_, *A*_2_, and *A*_3_ can be expressed in terms of these quantities as shown below:

$$R_{12}=\frac{\rho \left[1+\tau_{2}^{2}(1-2\rho )\right]}{1-\rho^{2}\tau_{2}^{2}}\approx \rho \left[1+\tau_{2}^{2}\right],$$ (22)

$$T_{23}=\frac{{\left(1-\rho \right)}^{2}\tau_{2}}{1-\rho^{2}\tau_{2}^{2}}\approx [1-2\rho ]\tau_{2},$$ (23)

$$R=\tau_{1}R_{12}\tau_{1}\approx \tau_{1}^{2}\rho \left[1+\tau_{2}^{2}\right],$$ (24)

$$T=\tau_{1}T_{23}\tau_{3}\approx \tau_{1}\tau_{2}\tau_{3}[1-2\rho ],$$ (25)

$$A_{1}=\left[1-\tau_{1}\right]\left[1+\tau_{1}R_{12}\right],$$ (26)

$$A_{2}=\tau_{1}\left[1-R_{12}+T_{23}\right],$$ (27)

$$A_{3}=\tau_{1}T_{23}\left[1-\tau_{3}\right].$$ (28)

The approximations on the right hand side of Eq. (22) and Eq. (23) conserve energy, are accurate to order *ρ* ^2^, and will be useful when *ρ* ^2^ << 1. These approximations could also have been derived directly from ray tracing.

The extension of this type of analysis to three or more reflecting boundaries for all powers of *ρ* probably becomes impractically complex. On the other hand, ray tracing to generate all terms linear in *ρ* appears relatively straightforward. But, the result will be useful only when the interference effects are small compared to the effects of interest.

## 4. Examples

**Fig. 2:**
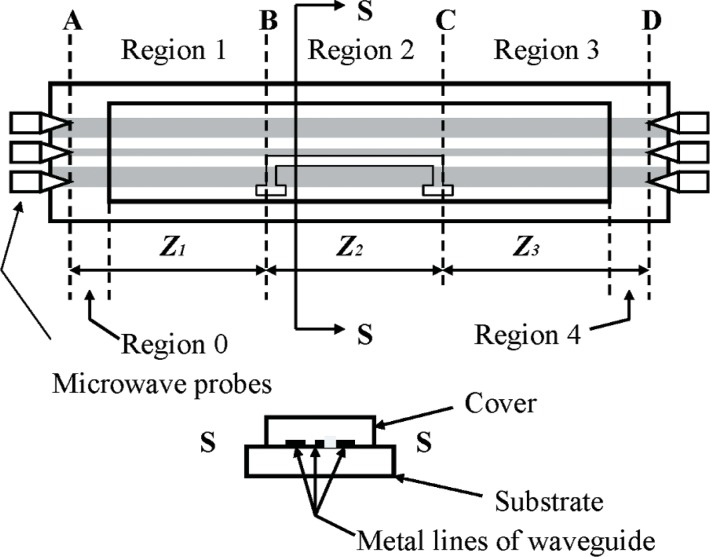
Top and cross-sectional views of a co-planar transmission line comprised of a cover over metal lines deposited on a substrate.

Reference [2] describes in detail an application of the equations given above; including a comparison of the derived results with experimental measurements of microwave heating of water in a microchannel. Figure 2 shows a top and a side view of the coplanar-waveguide transmission line of [2]. It is comprised of metal lines on a borosilicate glass substrate and a rectangular poly-dimethylsiloxane (PDMS) cover attached to the transmission line by contact adhesion. Region 1 of the transmission line extends from the microwave probes on the left side of the transmission line to the left end of the microchannel. Region 2 extends from the left end to the right end of the microchannel. Region 3 extends from the right end of the microchannel to the microwave probes on the right side of the transmission line. Regions 0 and 1 extend from the microwave probes to the closest end of the cover, respectively.

**Fig. 3:**
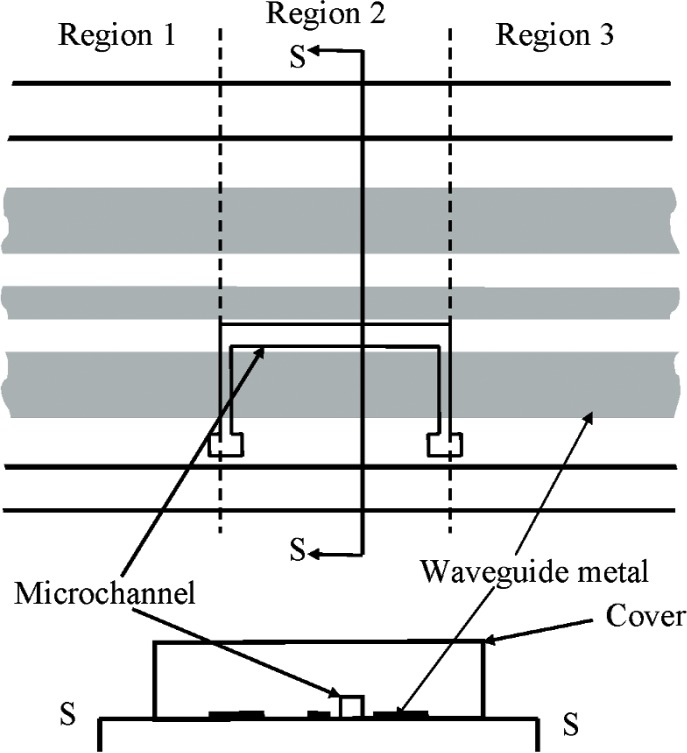
Close up view of the removable cover of Fig. 2 showing that the cover contains a microchannel.

**Fig. 4:**
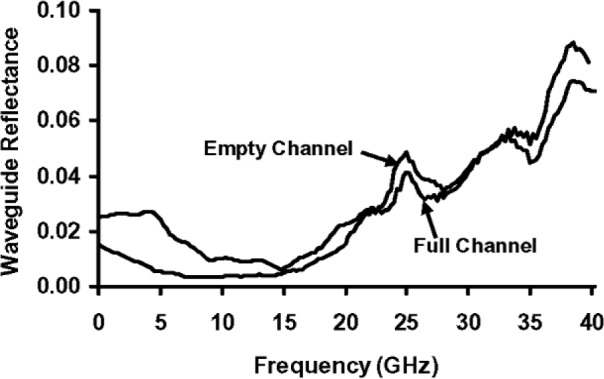
Reflectance spectra of the microfluidic transmission line described in [2].

**Fig. 5:**
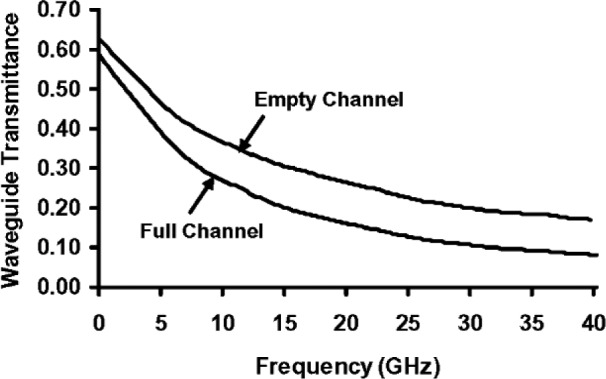
Transmittance spectra of the microfluidic transmission line described in [2].

Figure 3 is a closer view of the part of Fig. 2 that shows the microchannel in the cover over the coplanar waveguide. Figure 3 is not to scale, and the channel cross section, which is actually trapezoidal, is only shown schematically. Figure 4 plots the transmission-line reflectance spectra measured with a vector network analyzer, as described in Ref. [2], both when the microchannel in Fig. 3 was empty, and when it was full of de-ionized (DI) water. Figure 5 plots the transmittance spectra for the same configurations of the transmission line. Most of the features on the reflectance spectra are probably interference fringes caused by multiple reflections between the boundaries of the homogeneous regions in the transmission line. Any interference fringes present in the transmission data are much less pronounced than on the reflection data due to the different scale on the y-axes in these two figures.

The metal lines in the transmission line were 500 nm thick, vacuum-deposited gold on thin chromium adhesion layers, *z*_1_ ≈ *z*_3_ ≈ 0.569 cm, and *z*_2_ ≈ 0.362 Over the frequency range of Figs. 4 and 5 borosilicate glass and PDMS are approximately lossless (do not absorb microwave power) and have dielectric constants not very different from air when compared with those of gold and water. Therefore, the first assumption needed to apply the above equations to the transmission line of Fig. 2 is that the transmission line properties in Regions 0, 1, 3, and 4 in Fig. 2 are identical. This is illustrated by showing Region 0 as a subset of Region 1 and Region 4 as a subset of Region 3 in Fig. 2. Thus, the three-region transmission line in Fig. 1 approximates the five-region transmission line in Fig. 2 for the analysis presented here.

**Fig. 6:**
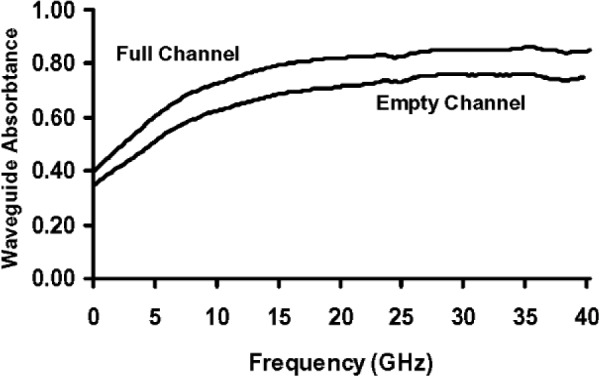
Absorption spectra of the microfluidic transmission line described in [2].

Figure 6 plots the empty- and full-channel absorption spectra of [2]

$$A_{e}=1-R_{e}-T_{e},$$ (29)

$$A_{f}=1-R_{f}-T_{f},$$ (30)

respectively, where subscript *e* refers to the empty-channel data, and subscript *f* refers to the full-channel data. Any interference fringes on the empty and full-channel absorption spectra below about 25 GHz are very small compared to the difference between the two spectra. This fact suggests that the approximate equations derived here, which ignore interference effects, can be used to calculate the fraction of the incident microwave power that is absorbed in the channel water with little error due to interference effects below this frequency. Features that may be interference fringes are more evident above 25 GHz, but they are still relatively small compared to the difference between the absorption spectra, so the errors incurred will be relatively small, even in this spectral region.

### 4.1 Difference in Absorbed-Power Fractions

The simplest estimate of the fraction of the microwave power incident on the filled transmission line that is absorbed in the water in the microchannel is the difference between the fraction of the power absorbed when the channel is full and when it is empty, which is given by

$$\Delta A=A_{f}-A_{e}=R_{e}+T_{e}-R_{f}-T_{f}.$$ (31)

However, as will be shown below, this is only a lower bound to the fraction of the power absorbed in the microchannel water and can greatly underestimate the actual fraction absorbed.

### 4.2 Reflection Only From the Left Boundary of Region 1

To derive a more accurate approximation, further assumptions about the transmission line are required. For instance, it can be assumed that Eqs. (1–5) describe the empty device. In other words, *R*_12_ = *R*_23_ = *R*_3_ = 0 and *ρ* ≠ 0 is the power reflection coefficient at the left boundary of Region 1. Let *R* = *R_e_* and *T* = *T_e_* be the measured reflectance and transmittance for the empty transmission line in Eqs. (1) and (2). In this case, these equations can be combined to give the attenuation coefficient of the empty transmission line as

$$\alpha_{m}=\frac{-1}{2\left[z_{1}+z_{2}+z_{3}\right]}\ln\left(\frac{T_{e}}{1-R_{e}}\right),$$ (32)

and *A*_1_*_em_*, *A*_2_*_em_*, and *A*_3_*_em_* can be calculated from Eqs. (3–5), and Eq. (32), with *α*_1_ = *α*_2_ = *α*_3_ = *α_m_*. Here the numeric subscripts designate the region, subscript *e* designates the empty transmission line, and subscript *m* designates absorption due to the metal conductors.

Now let *R* = *R_f_* and *T* = *T_f_* be the measured reflectance and transmittance calculated from the S-parameters when the channel is filled with DI water. The next assumption required is that the transmission-line attenuation coefficient due to the metal conductors, does not change when the microchannel in the transmission line is filled with water. This is a good approximation, provided that the addition of the water does not substantially change the field distribution on the surface of the metal conductors, even if it changes the distribution significantly in the middle of the channel. Regions 1 and 3 are still identical, and *α*_1_ = *α*_3_ = *α_m_*, but *α*_2_ = *α_m_* + *α_w_*, where *α_w_* is the portion of the attenuation coefficient of the transmission line in Region 2 due to the additional absorption by the water in the channel. In this case, Eqs. (1) and (2) can be combined to give

$$\alpha_{w}=\frac{-1}{2z_{2}}\left\{\ln\left(\frac{T_{f}}{1-R_{f}}\right)-2\alpha_{e}\left[z_{1}+z_{2}+z_{3}\right]\right\}$$ (33)

and *A*_1_*_fm_*, *A*_2_*_fm_*, *A*_2_*_fw_*, and *A*_3_*_fm_* can be calculated from Eqs. (3–5), Eq. (32), and Eq. (33) with *α*_1_ = *α*_3_ = *α_m_* and *α*_2_ = *α_m_* + *α_w_*. Again, the numeric subscripts designate the region, but subscript *f* designates the filled transmission line, and subscript *w* designates absorption due to the water in the microchannel.

Table 1 presents the results of applying Eqs. (32) and (33) to S-parameter data measured at 5.02 GHz [1]. The first and second sections of the table compare the measured and calculated parameters for the empty transmission line and the transmission line filled with DI water, respectively. First, notice that the calculation conserves power in that the sum of the reflected, transmitted, and absorbed fractions of the incident power is unity. Also note that *R_f_* ≠ *R_e_* shows at least one parameter changed when the transmission line was filled with water, and explains why *A*_1_*_f_* ≠ *A*_l_*_e_* in Table 1.

Also in Table 1, notice that *T_f_* ≠ *T_e_* and *A*_2_*_f_* ≠ *A*_2_*_e_*. A small fraction of this difference is attributable to violations of the assumptions on which the analysis was based. The main contributor is the large difference between the attenuation coefficients *α*_2_ = *α_m_* for the empty channel case, and *α*_2_ = *α_m_* + *α_w_* when the channel is filled with water. In other words, filling the microchannel with water has doubled the absorption (attenuation) in Region 2, with about half of the power being absorbed in the metal conductors and the other half being absorbed in the water in that region.

More precisely, the fraction *A*_2_*_fm_* of the power absorbed in the Region 2 metal of the full-channel transmission line is only about 93 % as large as the fraction *A*_2_*_em_* absorbed in the empty-channel transmission line. This difference is not the result of experimental error or a violation of the assumptions used in deriving the model. Instead, this difference is to be expected. When the water is present, the microwave power decreases more rapidly with distance along the channel than when the channel is empty due to the addition of water absorption to metal absorption. Therefore, there is less power available for the metal in Region 2 to absorb when the channel is full than when it is empty. In other words, water absorption competes with metal absorption in Region 2 when the channel is full of water.

**Fig. 7:**
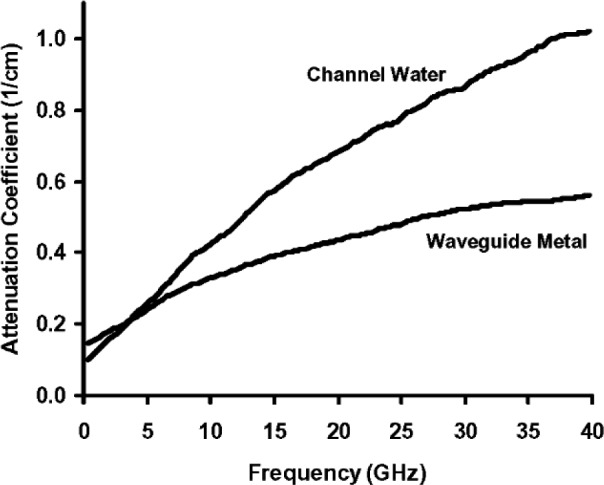
Microwave attenuration coefficient data for the metal conductors and the channel water for the transmission line described in [2].

Figure 7 compares the amplitude-attenuation coefficients for the metal conductors *α_m_* and the channel water *α_w_* that were calculated from the measured reflectance and transmittance spectra as illustrated in Table 1. Inspection of Fig. 7 reveals that interference fringes are evident in both spectra, but are most obvious in the *α_w_* spectrum. These are almost certainly errors caused by ignoring interference among multiple reflections in the approximate equations. But, these errors are small compared to the major trends in the spectra.

### 4.3 Alternative Assumptions and Comparison of Results

The model given in Eq. (31) of Sec. 4.1 can be rewritten as

$$A_{2fw}=\Delta A$$ (34)

in terms of the definitions given in connection with Table 1, where Δ*A* is defined in Eq. (31). Recall that Eq. (31) was derived by ignoring the competition between absorption by Region 2 water and metal.

For comparison with other models considered in this paper, the model of Sec. 4.1 will be referred to as Model (a). Similarly, the model discussed in Sec. 4.2 based on the assumption that only the reflection coefficient at the left probe is non-zero, will be referred to as Model (c). As alternatives to these models, the reflection coefficients might be assumed to be

(b) zero because they are small,
(d) non-zero only at the left end of the micro-channel,
(e) non-zero only at both probes,
(f) non-zero only at both ends of the microchannel,
(g) non-zero only at the right end of the micro-channel,
(h) non-zero only at the right probe.

These possibilities will be considered next. For case (b), which assumes that all reflection coefficients are zero, the same equations used in Sec. 4.2 can be used with the assumption that *R_e_* = *R_f_* = 0. For case (h), Eqs. (1) and (12) can be combined to eliminate *τ*_1_*τ*_2_*τ*_3_ in these equations with the result

$$\rho =\frac{2R+T^{2}-\sqrt{T^{4}+4RT^{2}}}{2R},$$ (35)

which can be substituted first into Eq. (32) to determine *α_m_* and then along with *α_m_* into Eq. (33) to determine *α_w_*.

Closed form solutions are not possible for the remaining equations, so they must he solved numerically. For case (d), which assumes non-zero reflection only at the left end of the microchannel, the approximations on the right hand side of Eqs. (7) and (1) can be combined to eliminate *ρ* to give,

$$\frac{R}{\tau_{1}\tau_{2}}+\frac{T}{\tau_{1}\tau_{2}\tau_{3}}=1,$$ (36)

which can then be solved numerically, first, for *α_m_* in terms of *R_e_* and *T_e_* and then for *α_w_* in terms of *α_m_*, *R_w_*, and *T_w_*.

For case (g), which assumes non-zero reflection only at the right end of the microchannel, the approximations on the right hand side of Eqs. (9) and (1) can be combined to eliminate *ρ* to give,

$$\frac{R}{\tau_{1}^{2}\tau_{2}^{2}}+\frac{T}{\tau_{1}\tau_{2}\tau_{3}}=1,$$ (37)

which can then be solved numerically, first, for *α_m_* in terms of *R_e_* and *T_e_* and then for *α_w_* in terms of *α_m_*, *R_w_*, and *T_w_*.

For case (e), which assumes non-zero reflection only at both probes, the approximations on the right hand side of Eqs. (16) and (17) can be combined to eliminate *ρ* to give,

$$\frac{2R}{1+\tau_{2}^{2}}+\frac{T}{\tau_{1}\tau_{2}\tau_{3}}=1,$$ (38)

which can then be solved numerically, first, for *α_m_* in terms of *R_e_* and *T_e_* and then for *α_w_* in terms of *α_m_*, *R_w_*, and *T_w_*.

For case (f), which assumes non-zero reflection only at both ends of the microchannel, the approximations on the right hand side of Eqs. (24) and (25) can be combined to eliminate *ρ* to give,

$$\frac{2R}{\tau_{1}^{2}\left[1+\tau_{2}^{2}\right]}+\frac{T}{\tau_{1}\tau_{2}\tau_{3}}=1,$$ (39)

which can then be solved numerically, first for *α_m_* in terms of *R_e_* and *T_e_* and then for *α_w_* in terms of *α_m_*, *R_w_*, and *T_w_*.

Table 2 lists values of *R_e_* and *T_e_* obtained at 5 GHz, 15 GHz, and 23 GHz [1, 2]. Table 3 compares the results of applying the different models derived above to the data in Table 2. The approximation *A*_2_*_fw_* = Δ*A* of Eqs. (31) and (34), which is the simplest estimate of the fraction of the microwave power absorbed in the water, is also shown in the table for comparison. Notice that this estimate is significantly smaller at all three frequencies than that based on the other approxi mations discussed in this paper. This is a general result because Δ*A* completely ignores the competition between the water and metal absorption processes, which reduces the total amount of power absorbed in the metal when the water is present, as discussed previously.

The data at different frequencies presented in Table 3 illustrate different relations among the empty- and full-channel transmission-line reflectances *R_e_* and *R_f_*. The values of *R_e_* and *R_f_* are given in Table 2, but they are also given by *ρ_e_* and *ρ_f_* under (c) in Table 3, because this is the case where the only reflection occurs at the left probe. Relatively speaking, at 5 GHz, *R_e_* is large (≈ 0.025) while *R_f_* is small (≈ 0.005) and *A*_2_*_f w_* is small (≲ 0.15). At 23 GHz, *R_e_* and *R_f_* are large, *R_f_* − *R_e_* is small, and *A*_2_*_f m_* is large (≳0.15). At 15 GHz, both *R_e_* and *R_f_* and their difference are small, but *A*_2_*_f w_* is large.

**Fig. 8:**
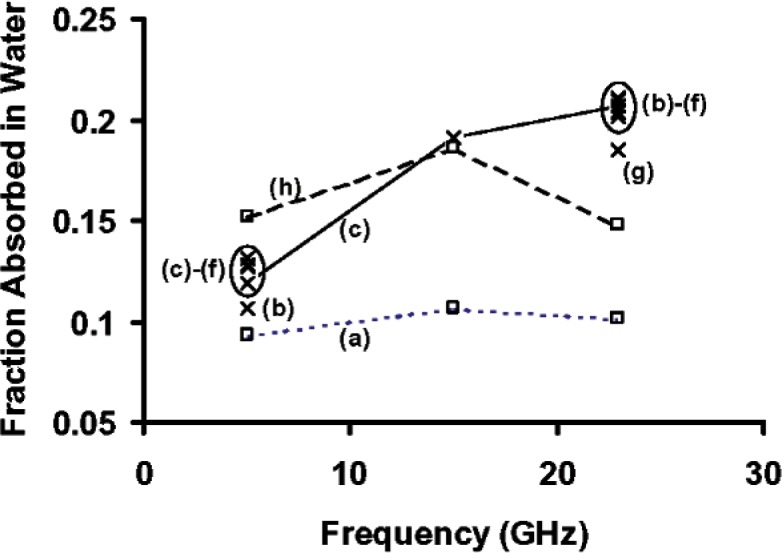
Comparison of the fraction of the incident microwave power absorbed by the water in the channel in the transmission line described in [2]. Models (a) - (h) are described in the text.

Figure 8 compares the fraction *A*_2_*_f w_* of the incident power that is absorbed in the microchannel water as calculated with the different models. Models (a) and (h) are plotted with squares, and the other models are plotted with times signs. The data points for (a) and (h) are connected with dashed lines, and the data points for (c) are connected with solid lines. An ellipse encircles a cluster of data points at 5 GHz, and another ellipse encircles a cluster of data points at 23 GHz. The cluster is so tight at 15 GHz that no ellipse is needed. The models that have at least one non-zero reflection coefficient on the left side of the microchannel (c - f) agree among themselves, significantly better than the other models. Also (b), which is based on assuming all reflection coefficients to be zero, agrees much better with these than do the models that assume non-zero reflection coefficients only to the right side of the micro-channel. The reasons for and implications of these facts, are discussed in the next section.

## 5. Discussion and Conclusion

Equations that describe the absorption of microwave power in the lossy components of a complex transmission line have been derived for a number of simplified models of the general three-region transmission line. Examples of the use of these equations to estimate the fraction of the power absorbed in the water in a microchannel embedded in a transmission line were described. These examples required three assumptions common to all of the examples. First, it was assumed that each transmission line could be divided into three homogeneous regions extending from the probes on one side of the device to the probes on the other side of the device. Second, it was assumed that the metal conductors and channel water are the only lossy components of the transmission line. Third, it was assumed that the portion of the attenuation coefficient of the transmission line that describes absorption of microwave power in the metal conductors did not change when the channel was filled with water. This is a good assumption if the presence of the water does not significantly change the field distribution on the surface of the metal conductors.

For some of the examples, it was further necessary to assume that the reflections at three of the four region boundaries in the transmission line were zero, even though what appeared to be interference fringes on the transmission line reflectance spectra suggest that was not the case. The results derived for these examples are rigorous. For two of the examples, (e) and (f), it was assumed that reflections at two of the four discontinuities were zero. These models are the most realistic because they assume identical reflection coefficients at pairs of boundaries that are mirror images of each other and should therefore be identical in theory, and similar in practice. On the other hand, the results based on these models are accurate only to the extent that interference effects are negligible, and then only to order *ρ*^2^, which will be more than adequate in many applications.

At the three frequencies for which data are reported in Fig. 8, the results of four of the models (c - f) form tight clusters. Ellipses encircle these clusters at 5 GHz and 23 GHz, while the data in the cluster at 15 GHz cannot be resolved at the scale of the figure. Model (b) gives results that agree much better with the data in the clusters than do (a) and (h). It is reassuring in this connection that (a) and (h) are the least realistic models of the transmission line shown in Fig. 2.

It has already been mentioned that (a) ignores the effect of the competition between the absorption by water and the metal conductors, and it only provides a lower bound to the fraction of the power absorbed in the water. For the transmission line analyzed here, (h) requires an unrealistically large reflection coefficient at the right-hand probe to compensate for the absorption that is associated with two complete passes along the transmission line. To a lesser extent, (g) also suffers from this problem.

The fact that five of the eight models compared here give substantially identical results for the fraction of the incident microwave radiation that is absorbed in a water filled channel under a range of reflection conditions suggests that these models can be used with some confidence. Specifically, they should provide reasonably accurate results when the measured reflectance as well as the interference fringes on the measured reflectance and transmittance spectra, are small compared to the absorbed fraction of interest. The reason for this is, that the fraction of the microwave power absorbed in a transmission-line region as calculated from these models is not very sensitive to the detailed assumptions about which boundaries are reflecting. On the other hand, the calculated values of the reflection coefficients are quite sensitive to the same assumptions. Nevertheless, if it is not obvious from physical reasoning or examination of experimental reflection and transmittance spectra which of these reflection models best describes a particular complex transmission line, then it is probably a good idea to compare the results of a number of models, as was done here.

In the general case, three-region devices will have four non-zero, non-identical reflection coefficients. Similar approximate equations that are accurate when the products of all combinations of two of the power reflection coefficients are small, can be easily derived for this case, but more independent measurement results will be needed to use these equations as illustrated here. The results could be obtained by performing the S-parameter measurements with the same PDMS cover located in different positions along the axis of the coplanar waveguide to change the dimensions of *z*_1_ and *z*_3_. This will change the fractions of the power returned to Boundary A by the reflections from Boundaries B, C, and D, and will change them in different ways for each boundary.

## Figures and Tables

**Table 1:** Measured and calculated parameters for the device described in [1] at 5.02 GHz. The subscript numbers correspond to the different regions. The subscripts *e* and *f* correspond to an empty channel and a channel filled with water, respectively, and the subscripts *m* and *w* correspond to the metal conductors and channel water

Condition	Parameter	Value	Unit
Empty Channel	*S*_11_*_e_*	−15.9956	dB
	*S*_21_*_e_*	−3.24646	dB
	*R_e_*	0.025144	
	*T_e_*	0.473537	
	*α_m_*	0.240687	cm^−1^
	*A*_1_*_em_*	0.233569	
	*A*_2_*_em_*	0.118546	
	*A*_3_*_em_*	0.149204	
	*R_e_*+*T_e_*+*A*_1_*_em_*+*A*_2_*_em_*+*A*_3_*_em_*	1.000000	

Filled Channel	*S*_11_*_f_*	−22.6425	dB
	*S*_21_*_f_*	−3.97558	dB
	*R_f_*	0.005442	
	*T_f_*	0.400352	
	*α_w_*	0.259523	cm^−1^
	*A*_1_*_fm_*	0.238289	
	*A*_2_*_fm_*	0.110560	
	*A*_2_*_fw_*	0.119212	
	*A*_3_*_m_*	0.126145	
	*R_f_*+*T_f_*+*A*_1_*_fm_*+*A*_2_*_fm_*+*A*_2_*_fw_*+*A*_3_*_fm_*	1.000000	

**Table 2:** Measured transmittance-line reflectance and transmittance at three frequencies

Frequency	*R_e_*	*T_e_*	*R_f_*	*T_f_*
5 GHz	0.025144	0.473537	0.005442	0.400352
15 GHz	0.006318	0.308259	0.004613	0.203731
23 GHz	0.028247	0.244598	0.028454	0.142578

**Table 3:** Comparison of results from the different models discussed. The results in the column headed by *ρ_l p_* are based on the assumption that the reflection coefficient at the left probe was non-zero. Similarly, the results in the column headed by *ρ_r c_* are based on the assumption that the reflection coefficient at the right end of the channel is non-zero, etc. The other quantities are defined in the text.

Condition	Parameter	Δ*A* (a)	all zero (b)	Non-Zero Reflection Coefficients					
				*ρ_l p_* (c)	*ρ_l c_* (d)	*ρ_l p_* =*ρ_r p_* (e)	*ρ_l c_* = *ρ_r p_* (f)	*ρ_r p_* (g)	*ρ_r p_* (h)
5 GHz Empty Channel	*ρ_e_*		0.0000	0.0251	0.0429	0.0202	0.0249	0.0590	0.0924
	*α_m_*		0.2492	0.2407	0.2346	0.2354	0.2322	0.2289	0.2169
	*A*_2_*_em_*		0.1243	0.1185	0.1145	0.1186	0.1183	0.1236	0.1190
5 GHz Full Channel	*ρ_f_*		0.0000	0.0054	0.0093	0.0047	0.0063	0.0194	0.0318
	*α_w_*		0.2319	0.2595	0.2789	0.2759	0.2850	0.2888	0.3211
	*A*_2_*_fm_*		0.1147	0.1106	0.1076	0.1087	0.1074	0.1079	0.1029
	*A*_2_*_fw_*	0.0929	0.1068	0.1192	0.1279	0.1274	0.1319	0.1191	0.1524
15 GHz Empty Channel	*ρ_e_*		0.0000	0.0063	0.0152	0.0058	0.0097	0.0263	0.0589
	*α_m_*		0.3928	0.3902	0.3871	0.3884	0.3858	0.3834	0.3720
	*A*_2_*_em_*		0.1582	0.1569	0.1549	0.1569	0.1568	0.1598	0.1576
15 GHlz Full Channel	*ρ_f_*		0.0000	0.0046	0.0111	0.0044	0.0089	0.0426	0.0917
	*α_w_*		0.5720	0.5744	0.5778	0.5757	0.5742	0.5488	0.5230
	*A*_2_*_fm_*		0.1309	0.1298	0.1283	0.1296	0.1292	0.1333	0.1325
	*A*_2_*_fw_*	0.1062	0.1907	0.1911	0.1916	0.1922	0.1923	0.1908	0.1862
23 GHz Empty Channel	*ρ_e_*		0.0000	0.0282	0.0773	0.0265	0.0495	0.1354	0.2591
	*α_m_*		0.4694	0.4598	0.4425	0.4512	0.4346	0.4209	0.3694
	*A*_2_*_em_*		0.1689	0.1631	0.1529	0.1635	0.1624	0.1789	0.1673
23 GHz Full Channel	*ρ_f_*		0.0000	0.0285	0.0779	0.0278	0.0641	0.2791	0.4396
	*α_w_*		0.7455	0.7452	0.7446	0.7416	0.7001	0.4944	0.3599
	*A*_2_*_fm_*		0.1325	0.1279	0.1198	0.1278	0.1262	0.1579	0.1517
	*A*_2_*_fw_*	0.1018	0.2104	0.2072	0.2015	0.2101	0.2032	0.1855	0.1478
